# Integrated Care of Atrial Fibrillation Using the ABC (Atrial fibrillation Better Care) Pathway Improves Clinical Outcomes in Chinese Population: An Analysis From the Chinese Atrial Fibrillation Registry

**DOI:** 10.3389/fcvm.2021.762245

**Published:** 2021-11-18

**Authors:** Yu-Feng Wang, Chao Jiang, Liu He, Xin Du, Cai-Hua Sang, De-Yong Long, Ri-Bo Tang, Jian-Zeng Dong, Gregory Y. H. Lip, Chang-Sheng Ma

**Affiliations:** ^1^Department of Cardiology, Beijing AnZhen Hospital, Capital Medical University, National Clinical Research Centre for Cardiovascular Diseases, Beijing, China; ^2^Heart Health Research Centre, Beijing, China; ^3^The George Institute for Global Health, Faculty of Medicine, University of New South Wales, Sydney, NSW, Australia; ^4^Centre for Cardiovascular Diseases, The First Affiliated Hospital of Zhengzhou University, Zhengzhou University, Henan, China; ^5^Liverpool Centre for Cardiovascular Science, Liverpool Heart and Chest Hospital, University of Liverpool, Liverpool, United Kingdom

**Keywords:** atrial fibrillation, ABC pathway, mortality, integrated care, comorbidity

## Abstract

**Background:** “Atrial fibrillation Better Care” (ABC) pathway has been proposed to improve the management of patients suffered from atrial fibrillation (AF). This integrated or holistic management approach comprise of three aspects, including “A” Avoid stroke or Anticoagulation; “B” Better symptom control with rate or rhythm control strategies; “C” Cardiovascular risk factor and Concomitant diseases management. We aimed to confirm the beneficial evidence of ABC pathway compliance in a Chinese AF cohort.

**Method and Results:** From the Chinese Atrial Fibrillation registry (CAFR) dataset, a total of 19,187 non-valvular AF patients were enrolled, of which 4.365 (22.8%) were ABC pathway compliant (ABC compliance group). During a median follow-up of 4.1 ± 1.8 years, The incident rate of all-cause death in ABC compliance group and non-ABC compliance group is 2.7 and 1.1 per 100 person-year (*p* < 0.001), the incident rate of ischemic stroke is 1.3 and 0.8% per 100 person-year (*p* < 0.001), the incident rate of composite outcome, which consist of all-cause death, ischemic stroke and intracranial hemorrhage, is 3.8 and 1.9 per 100 person-year (*p* < 0.001). On Cox multivariable analysis, ABC pathway shows an independently association with reduction of all-cause death [hazard ratio (HR) = 0.82; 95% confidence interval (CI) = 0.70–0.95] and the composite outcome (HR 0.86, 95% CI 0.76–0.96). The increasingly components of ABC integrated care compliance is associated with lower risk of all-cause death and composite events.

**Conclusion:** In a large cohort of Chinese AF patients, ABC pathway compliance shows an independently association with reduction of all-cause death and composite outcome of all-cause death, ischemic stroke and intracranial hemorrhage. Better compliance of ABC integrated care contributes to lower HR for adverse events.

## Introduction

Atrial fibrillation (AF), which is a highly prevalent arrhythmia, has contributed to substantial cardiovascular death and cardiac morbidity from AF-related complications (1). Indeed, AF is associated with higher all-cause mortality (4.6% per year), only 10% of death is caused by stroke and nearly 50–60% attribute to other cardiovascular events (2, 3). Hence a comprehensive and integrated care method to management of AF and its associated comorbidities has been proposed to reduce these adverse outcomes (4, 5).

The Atrial fibrillation Better Care (ABC) pathway was introduced as an method to streamline the integrated or holistic management of AF (6). The ABC pathway has three main components: “A” refers to Avoid stroke with Anticoagulation, which means. optimizing stroke prevention with oral anticoagulation (OAC); “B” involves Better symptom management,i.e. improvement on symptom with rate or rhythm control by patient-centered symptom directed decisions; “C” applies to Cardiovascular risk factor and other Concomitant diseases management, including lifestyle modification (6).

Several studies have shown that the ABC pathway compliant management could reducing risk of adverse events (7–12) and this integrated pathway approach has also been recommended by 2020 ESC AF guidelines (13). Nonetheless, relatively few studies have investigated the impact of ABC pathway compliance in Asian countries, with the evidence derived from one cluster randomized trial (14) and one cohort study from Korea (9).

In China, nearly 7.9 million (about 2%) people suffer from atrial fibrillation (15). In addition, the anticoagulated rate and comorbidities control rate are quite low (6 and 4% respectively) (15, 16), thus comprehensive management seems to be urgent for Chinese population. However, the beneficial evidence of this kind of pathway remains limited in Chinese population. In this analysis derived from the Chinese Atrial Fibrillation registry (CAFR) dataset, we investigated if ABC pathway compliance is associated with reduced adverse events in a large Chinese cohort consisting of AF patients.

## Materials and Methods

This retrospective analysis was based on the Chinese Atrial Fibrillation registry (CAFR), which is the largest, observational, prospective, register of AF patients in China. Detailed introduction about CAFR has been previously published (17). In short, the CAFR dataset has consecutively enrolled patients with AF from 31 tertiary and non-tertiary hospitals in Beijing, China, with regular follow-up of every 6 months for these patients. Pivotal data including demographic information, symptoms and signs related to AF, other comorbidities and related medication or procedures, physical examination and biochemical test results will be collected at baseline. Severe adverse events comprising the occurrence of death and other cardiovascular diseases will also be collected at each 6 month by specialized follow-up team and adjudicated by professional endpoints committee.

Patients who were older than 18 years and diagnosed by AF episode of more than 30 second documented by 12-lead electrocardiogram were qualified for inclusion. Exclusion criteria were listed as follows: (1) valvular AF, including any mechanical bioprosthetic valves, or moderate to severe mitral stenosis, (2) those who had no baseline data up to 6 months before enrollment, and (3) those who had missing data such as European Heart Rhythm Association (EHRA) symptom score. After all these selection procedures, we enrolled non-valvular AF patients amount to 19,187 in this ancillary study to confirm the beneficial evidence of the ABC compliance on clinical events of AF patients.

The ethics committees of Beijing Anzhen Hospital has approved CAFR study and all patients involved in this study were informed about the detailed information and signed up for participating consent form.

### Definition of the ABC Pathway Compliant Group

Based on the current clinical guidelines (13), the ABC pathway was defined as follows:

“A—Avoid stroke”— patients with a CHA_2_DS_2_-VASc score of 0 in men or 1 in women not receiving OAC and those with a CHA_2_DS_2_-VASc score of at least 2 in men or 3 in women anticoagulated were considered as compliant with the “A” criterion. We also considered patients with a CHA_2_DS_2_-VASc score of 1 in men or 2 in women as “A” criterion compliant, no matter whether OAC were used. Others were recognized as “non-compliant” with the “A” criterion.

“B—Better symptom control”— Patients with no symptoms or mild symptoms having no influence on their daily life will be considered as EHRA I or II level. So “B compliance” refers to patients with EHRA score of I or II. Paitents with EHRA score of III or IV will be categorized as “non B compliance”.

“C—Cardiovascular risk factor and other concomitant diseases optimization”— Cardiovascular diseases which is commonly associated with AF need to be treated with some specific medication or achieve standard goals. (1) for hypertension, baseline blood pressure ≤ 140/90 mmHg was considered as well-controlled. (2) for heart failure, we considered treatment with ACE inhibitors/angiotensin receptor blockers and beta-blockers; (3) for coronary artery disease, treatment with angiotensin-converting enzyme (ACE) inhibitors, beta-blockers and statins; (4) for peripheral artery disease, treatment with statins; (5) for previous stroke/transient ischaemic attack, treatment with statins; (6) for diabetes mellitus, a fasting blood glucose of less than 7.0 mmol/L or glycosylated hemoglobin of less than 6.5% at baseline was considered as well-controlled. “C compliance” implies that all of these related concomitant diseases were either well-controlled or treated with suitable medication or both.

Paitents were identified as ABC pathway compliance if all of the components of ABC were fulfilled.

### Outcomes Definition

The main events we assess in this study were all-cause motality, ischemic stroke, intracranial hemorrhage and a composite outcome of these three outcomes. Patients were followed from the index date until the study outcomes occurred or at end of follow-up, whichever occurred first. We compared these clinical events between AF patients with ABC pathway compliance (ABC compliance group) and those without this intergrated care (non-ABC compliance group).

### Statistical Analysis

Continuous variables were expressed as mean ± standard deviation and compared by Student's *t-*test. Categorical variables were reported as frequency (percentage) and evaluated by Fisher's exact test or Pearson's chi-square test. We mainly analyzed the comparisons of clinical events between patients with and without ABC compliance. We also examined the association between total number of ABC compliance fulfilled and clinical events. We presented the cumulative incidences of clinical events by Kaplan–Meier curves and compared across the ABC and non-ABC group with the log rank test. Cox multivariable regression model was employed to analyze the hazard ratios (HRs) for clinical outcomes according to ABC compliance and adjusted clinical variables in this model includes age, sex, BMI, AF-type, eGFR < 60, current smoking, RFCA, coronary artery disease, peripheral arterial disease, heart failure, hypertension, diabetes mellitus, and previous ischemic stroke/TIA. A two-side *p*-value of < 0.05 was considered significant. All Statistical analyses were performed using SAS programming version 9.4.

## Results

Among 19,187 patients enrolled in this study from CAFR dataset, 22.8% (*n* = 4,365) was defined as “ABC compliance group” and 77.2% (*n* = 14,822) was identified as “Non-ABC compliance group”. Baseline characteristic of these two groups were presented in Table 1.

**Table 1:** Baseline characteristics according to the ABC compliance.

	**All patients**	**ABC group**	**Non-ABC group**	***P*-value**
	***N =* 19,187**	***N =* 43,65**	***N =* 14,822**	
Age (years)	64.2 ± 12.0	62.4 ± 11.5	64.7 ± 12.1	<0.0001
Age≥75, *n* (%)	4,187 (21.8)	633 (14.5)	3,554 (24.0)	<0.0001
Female, *n* (%)	7,389 (38.5)	1,500 (34.4)	5,889 (39.7)	<0.0001
SBP(mmHg)	128.3 ± 16.6	120.0 ± 10.2	130.5 ± 17.5	<0.0001
Body mass index (kg/m^2^)	25.5 ± 3.5	25.5 ± 3.6	25.5 ± 3.5	0.0045
AF type, *n* (%)				
New onset	1,179 (6.1)	229 (5.3)	950 (6.4)	0.0096
Paroxysmal	10,982 (57.2)	2,490 (57.0)	8,492 (57.3)	
Persistent/permanent	7,026 (36.6)	1,646 (37.7)	5,380 (36.3)	
eGFR <60 (CKD-EPI), *n* (%)	1,876 (9.8)	224 (5.1)	1,652 (11.2)	<0.0001
Current Smoking, *n* (%)	2,253 (11.7)	602 (13.8)	1,651 (11.1)	<0.0001
RFCA (baseline), *n* (%)	8,644 (45.1)	1,910 (43.8)	6,734 (45.4)	0.0506
Comorbidities, *n* (%)				
Coronary artery disease	2,967 (15.5)	121 (2.8)	2,846 (19.2)	<0.0001
Peripheral arterial disease	153 (0.8)	10 (0.2)	143 (1.0)	<0.0001
Hypertension	12,250 (63.9)	2,231 (51.1)	10,019 (67.6)	<0.0001
Heart failure	2,719 (14.2)	187 (4.3)	2,532 (17.1)	<0.0001
Ischemic stroke or TIA	2,774 (14.5)	294 (6.7)	2,480 (16.7)	<0.0001
Diabetes mellitus	4,588 (23.9)	570 (13.1)	4,018 (27.1)	<0.0001
CHA_2_DS_2_-VASc, mean ± SD	2.5 ± 1.8	1.8 ± 1.3	2.7 ± 1.9	<0.0001
HAS-BLED, mean ± SD	1.8 ± 1.2	1.5 ± 0.9	1.9 ± 1.2	<0.0001
Medication, *n (%)*				
Warfarin	8,186 (42.7)	2,162 (49.5)	6,024 (40.6)	<0.0001
NOAC	4,150 (21.6)	749 (17.2)	3,401 (23.0)	<0.0001
ACEI/ARB	6,531 (34.0)	1,291 (29.6)	5,240 (35.4)	<0.0001
Beta-blocker	8,144 (42.5)	1,790 (41.0)	6,354 (42.9)	0.0288
Calcium channel blocker	852 (4.4)	178 (4.1)	674 (4.6)	0.1858
Statin	7,480 (39.0)	1,445 (33.1)	6,035 (40.7)	<0.0001
Aspirin or Clopidogrel	4,916 (25.6)	802 (18.4)	4,114 (27.8)	<0.0001
Insulin therapy or Oral antidiabetic drugs	2,290 (11.9)	309 (71)	1,981 (13.4)	<0.00001

*ABC, Atrial fibrillation Better Care; ACEI, angiotensin-converting-enzyme inhibitors; AF, Atrial Fibrillation; ARB, angiotensin receptor blockers; CHA2DS2-VASc, congestive heart failure, hypertension, age > 75 (doubled), diabetes mellitus, prior stroke or transient ischemic attack (doubled), vascular disease, age 65 to 74, female; eGFR, estimated glomerular filtration rate; HAS-BLED, uncontrolled hypertension, abnormal renal and/or hepatic function, stroke, bleeding history or predisposition, labile INR, elderly, drugs or excessive alcohol drinking; NOAC, non-vitamin K antagonist oral anticoagulant; SBP-systolic blood pressure; TIA, transient ischaemic attack*.

In comparison with non-ABC compliance group, the ABC compliance group were older (62.4 ± 11.5 vs. 64.7 ± 12.1, *p* < 0.001), less likely to be female (34.3 vs.38.7% *p* < 0.001) and had a lower systolic blood pressure (120.0 ± 10.2 vs. 130.5 ± 17.5, *p* < 0.001). The AF type in the ABC group was less likely to be new onset and paroxysmal AF. The ABC group also had a lower prevalence of comorbidities, including coronary artery disease, peripheral arterial disease, hypertension, heart failure, ischemic stroke or TIA, diabetes mellitus and chronic kidney disease (eGFR < 60), and were treated with less drugs.

As expected, the ABC group had a lower mean CHA_2_DS_2_-VASc score (1.8 ± 1.3 vs. 2.7 ± 1.9, *p* < 0.001) and HAS-BLED score (1.5 ± 0.9 vs. 1.9 ± 1.2, *p* < 0.001) than the Non-ABC group. There was a borderline significant difference in the proportion of catheter ablation use between these two groups (*p* = 0.0506).

### ABC Integrated Care Compliance

Specific compliance of patients involved in this study toward each components of the ABC care is shown in Table 2, as follows: 12,499 (65.1%) patients were managed in accordance with component “A”, followed by rules of OAC use from the international guidelines; the “B” criterion was fulfilled in 14,021 (73.1%) patients, who has no or only mild complain of clinical symptoms and was evaluated as EHRA I-II score due to the comprehensive management; “C” criterion-compliance, which refers to optimally treatment of associated concomitant diseases by specific medication or achievement of standard goals on the basis of international guidelines, was established in 9,347 (48.7%). The compliance varied among these comorbidities, from peripheral arterial disease, up to 61.4%, to coronary artery disease, only 25.0%.

**Table 2:** Compliance with the ABC pathway and its components.

**Study Groups**	**ABC Compliant**	**Non-compliant**
	**[*N* (%)]**	**[*N* (%)]**
A	12,499 (65.1)	6,688 (34.9)
B	14,021 (73.1)	5,166 (26.9)
C	9,347 (48.7)	9,840 (51.3)
Hypertension	6,034 (49.4)	6,189 (50.6)
Heart failure	562 (33.8)	1,101 (66.2)
Ischemic stroke or TIA	1,538 (55.4)	1,236 (44.6)
Diabetes mellitus	1,741 (43.5)	2,259 (56.5)
Coronary artery disease	740 (25.0)	2,219 (75.0)
Peripheral arterial disease	94 (61.4)	59 (38.6)
All ABC	4,365 (22.8)	14,822 (77.2)

*A, avoid stroke; B, better symptoms management; C, cardiovascular and other comorbidities; ABC, the full ABC pathway compliance; TIA, transient ischaemic attack*.

### Clinical Outcomes

During a median follow-up of 4.1 ± 1.8 years. ABC pathway performed a lower rates of all-cause death (2.7 vs. 1.1 per 100 person-years, *p* < 0.001), ischemic stroke (1.3 vs. 0.8 per 100 person-years, *p* < 0.001) and composite outcome (3.8 vs.1.9 per 100 person-years, *p* < 0.001) compared with no ABC compliance. Intracranial hemorrhage was not difference between these two groups (0.3 vs. 0.3 per 100 person-years, *p* = 0.8544) (Table 3).

**Table 3:** Outcomes according to compliance with the ABC pathway.

	**All patients**	**ABC group**	**Non-ABC group**	***P*-value**
	***N =* 19,187**	***N =* 4,365**	***N =* 14,822**	
All-cause mortality, n (100 person-year)	1,871 (2.3)	212 (1.1)	1,659 (2.7)	<0.0001
Stroke, n (100 person-year)	939 (1.2)	150 (0.8)	789 (1.3)	<0.0001
Intracranial hemorrhage, n (100 person-year)	213 (0.3)	49 (0.3)	164 (0.3)	0.8544
Composite outcome, n (100 person-year)	2,633 (3.4)	360 (1.9)	2,273 (3.8)	<0.0001

**Figure 1:**
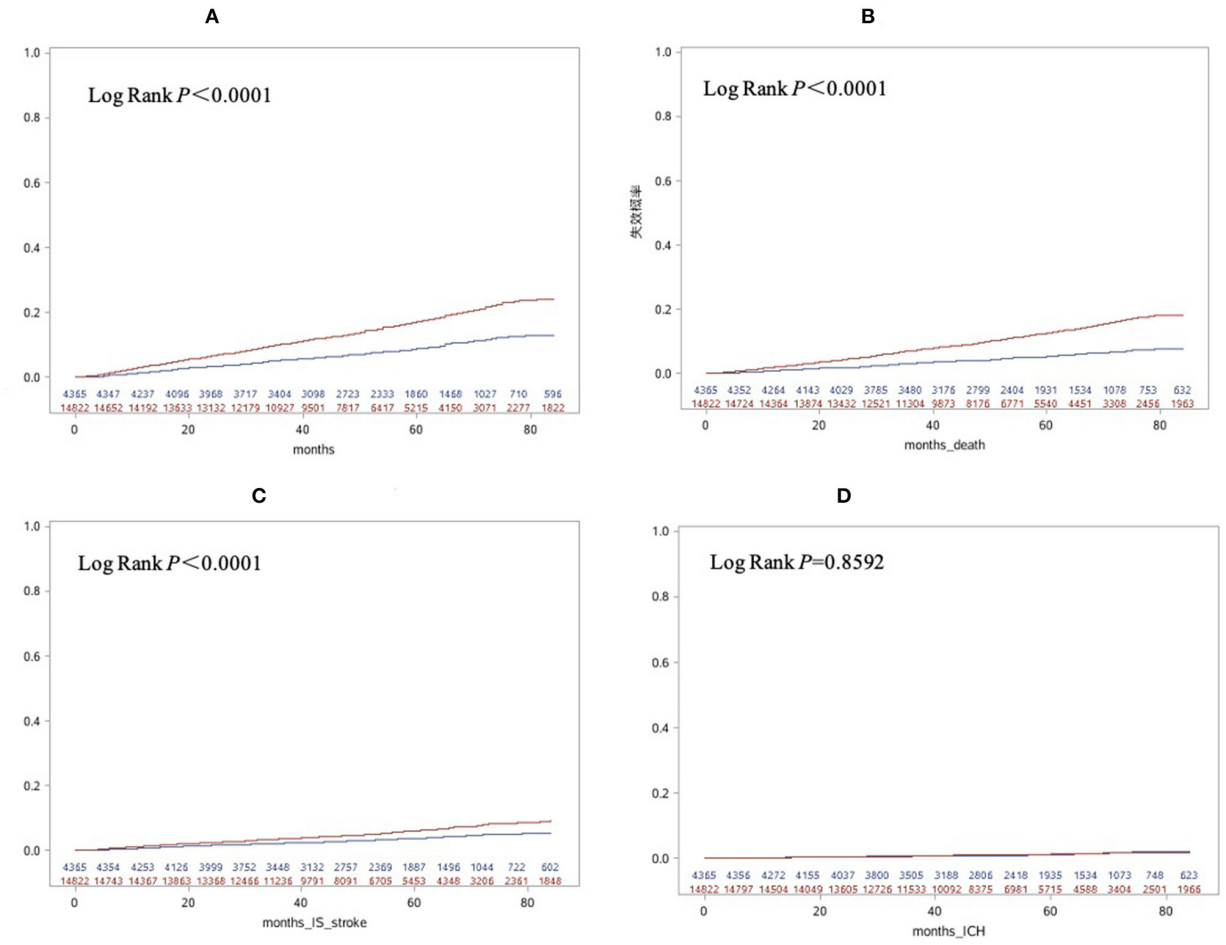
Cumulative incidences of composite outcome **(A)**, all-cause death **(B)**, stroke **(C)** and intracranial hemorrhage **(D)** according to compliance with integrated care based on the Atrial fibrillation Better Care [ABC] pathway. Blue line, ABC group; Red line, Non-ABC group.

The cumulative incidence of clinical outcomes including all-cause death, ischemic stroke and composite outcome were significantly lower in ABC compliance group compared with the non-ABC compliance group. No difference was found in the cumulative incidence of intracranial hemorrhage (Figure 1).

Cox multivariable regression analysis showed ABC compliance was independently associated with a lower risk of all-cause death (hazard ratio [HR], 0.82; 95% confidence interval [CI], 0.70–0.95) and composite outcome (HR, 0.86; 95% CI, 0.76–0.96). No significant association with stroke and intracranial hemorrhage were found in patients with ABC compliance care (HR, 0.87; 95% CI, 0.72–1.05 and HR, 1.22; 95% CI, 0.87–1.72, respectively) (Table 4).

**Table 4:** Regression models for the compliance of ABC in relation to outcomes.

	**Univariable**		**Multivariable**	
	**HR (95% CI)**	***p* Value**	**HR (95% CI)**	***p* Value**
All-cause mortality	0.41 (0.35, 0.47)	<0.0001	0.82 (0.70, 0.95)	0.0096
Stroke	0.61 (0.51, 0.73)	<0.0001	0.87 (0.72, 1.05)	0.1356
Intracranial hemorrhage	0.97 (0.70, 1.33)	0.8409	1.22 (0.87, 1.72)	0.2468
Composite outcome	0.50 (0.45, 0.56)	<0.0001	0.86 (0.76, 0.96)	0.0102

*Hazard ratios are adjusted for age, sex, BMI, AF-type, eGFR < 60, Current Smoking, RFCA, Coronary artery disease, Peripheral arterial disease, heart failure, hypertension, diabetes mellitus, and previous ischemic stroke/TIA*.

**Figure 2:**
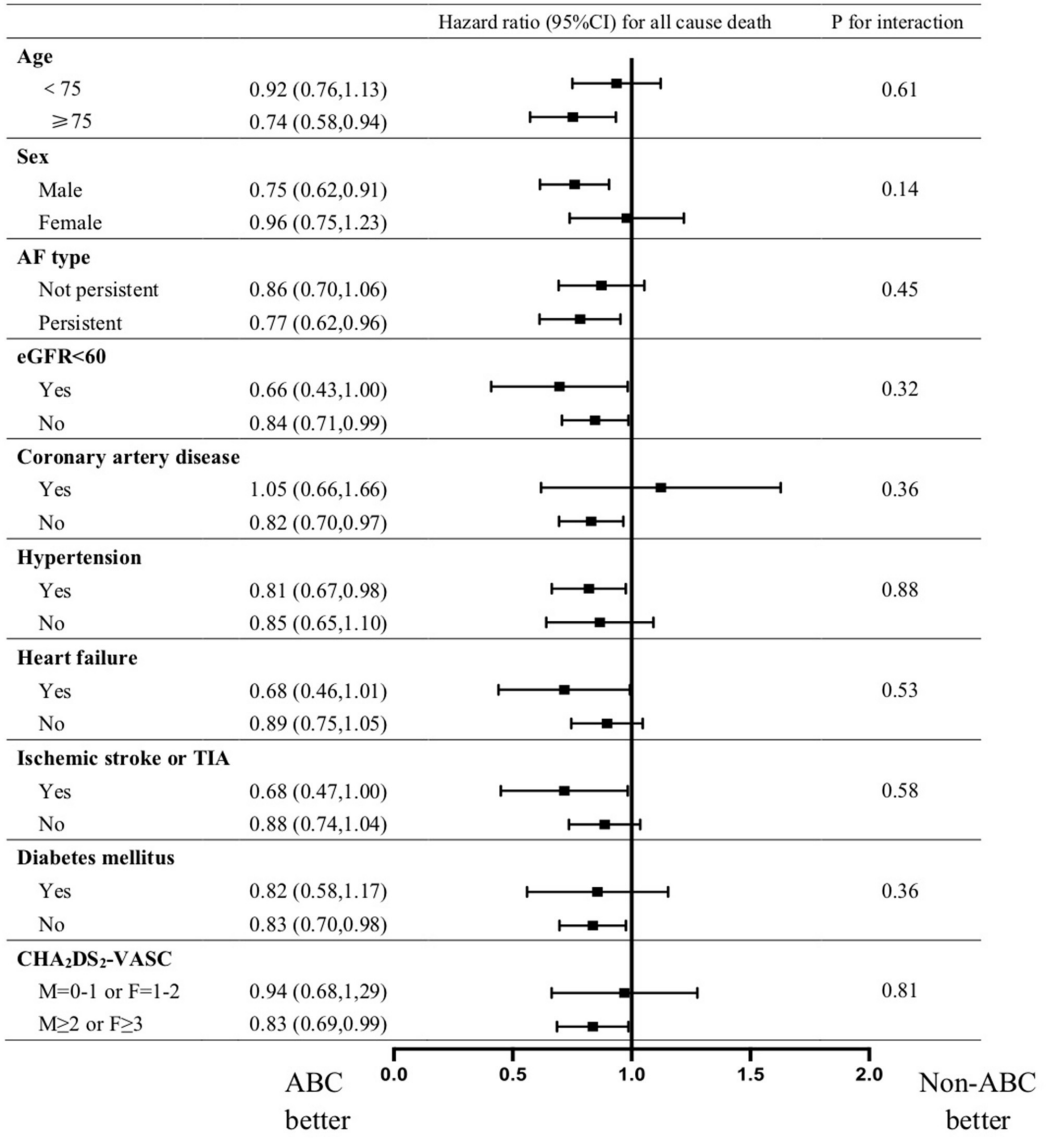
Forest plots presenting subgroup analysis for hazard ratio of all cause death for ABC compared with non-ABC group. Hazard ratios are adjusted for age, sex, BMI, AF-type, eGFR <60, Current Smoking, RFCA, Coronary artery disease, Peripheral arterial disease, heart failure, hypertension, diabetes mellitus, and previous ischemic stroke/TIA.

Subgroup analysis present a consistent result that ABC compliance is associated with a lower risk of all cause death in patients with AF regardless of age, sex, CHA_2_DS_2_-VASC score, AF-type,eGFR<60, coronary artery disease, peripheral arterial disease, heart failure, hypertension, diabetes mellitus, and previous ischemic stroke/TIA (Figure 2).

### Number of ABC Criteria Fulfilled and Clinical Outcomes

**Figure 3:**
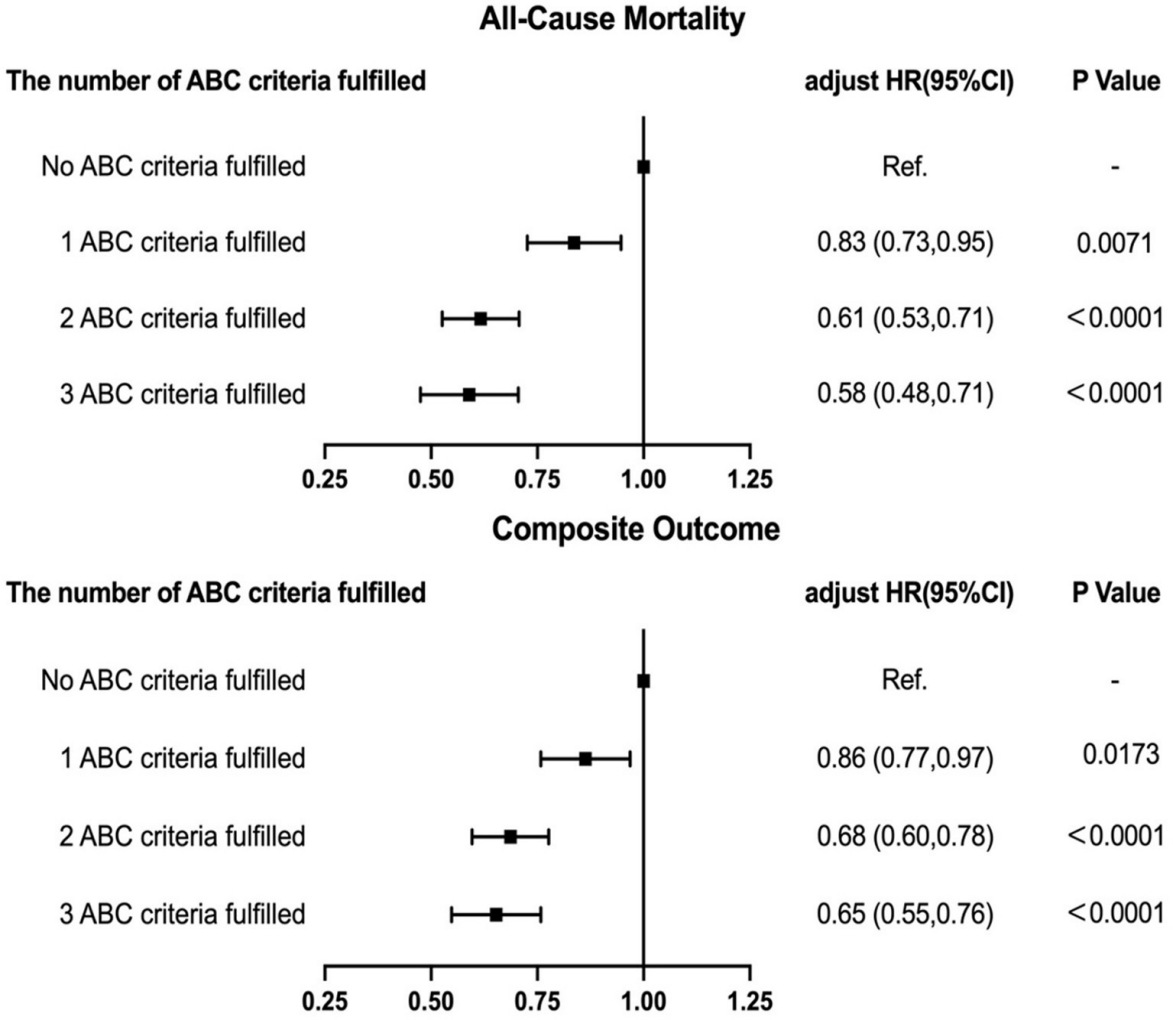
Relationship between total number of ABC criteria fulfilled and outcomes.

We examined the potential connection between fulfilled number of ABC component and adverse events. Adjusted Cox regression analysis showed that a higher numbers of ABC criteria fulfilled was independently associated with a progressively lower HRs of all-cause death and the composite outcome (Figure 3).

## Discussion

In this report from the CAFR Registry, we present novel data from a large observational cohort of Chinese AF patients, whereby clinical practice compliant with the ABC integrated care was associated with a significant lower risk in all-cause mortality and composite outcome of all-cause death, ischemic stroke and intracranial hemorrhage. Moreover, with more components of ABC pathway criteria fulfilled, the risk of all-cause mortality and composite outcome was progressively lower.

The favorable impact resulting from comprehensive care and integrated method to AF management is reported in a randomized control trial and various real-world evidence studies (18, 19), although relatively limited data come from large Asian cohorts. The ABC pathway, which provides a simple, effective, and easily operationalizable integrated pr holistic AF management for patients and their carers, satisfies these demand and was recommended by the 2020 European Society of Cardiology guidelines (13).

Previous studies have shown that therapies compliant with the holistic care (ABC compliance group) were associated with a significantly lower all-cause death and the composite outcome consisting of cardiovascular motality, myocardial infarction, ischemic stroke, total hospitalizations and major bleeding, compared with non-ABC compliant group (7–12). Also, a system review and a meta-analysis have revealed an obvious risk reduction in ABC group despite the variation including population selection, definition of ABC and clinical outcomes among these studies (20, 21). Our findings are consistent with the foregoing studies presenting lower hazard of all-cause death and the composite outcome in patients compliant with ABC care in comparsion to those without ABC pathway. The result that the risk of all-cause mortality and composite outcome was progressively lower with more components of ABC pathway criteria fulfilled was also found in other similar studies, which further points to an important lesson that a larger clinical benefit would accrue to those who were increasingly adherent to the ABC pathway management (7, 9, 10).

In our study, Cox regression did not present a significant lower risk in ischemic stroke and intracranial hemorrhage, and the similar result was also reported in other studies, including a recent randomized controlled trial (7, 10, 14). We hypothesized that these results may be caused by the relatively low incidence of stroke and intracranial hemorrhage events compared with other cohort studies, and the poor control of anticoagulation observed from Asian cohorts (22, 23) as nearly 50% of AF patients were prescribed warfarin in our study.

As the original cohort, from which the data were derived, represents the largest observational study for AF management in China, the results we presented are indicative of contemporary AF management nationwide in comparsion to previous studies exploring the beneficial evidence of ABC compliance approach that confined in limited cohorts, or those which were involved with patients from other countries (7–9). Moreover, this cohort comprises of the largest number of patients with ABC compliance management, presenting reliable results in relation to the amount of patients and events.

## Limitations

As an observational study, there are some limitations to be considered. Almost half of AF patients using warfarin in this cohort, and the lack of TTR data remains the efficiency of anticoagulation unknown, leading to some bias of the correlation of ABC adherent care and clinical outcomes. Even though the multivariable COX regression analysis presented an significant association between ABC compliance and reduced risk of these adverse events, some other related factors were not involved, including the presence of other concomitant diseases not available at baseline in this study or mentioned as part of the “C” criterion. Also, we have limited information on individual adherence and persistence to medication as well as the lack of a formalized approach to the evaluation of pharmacological treatment.

Finally, given the nature of the observational study, our data could only describe associations rather than demonstrate causality. Even if a large number of covariates have been adjusted in the the multivariable analysis, it seems to be unreasonable to make an inference of causality with the differences at baseline characteristics between the ABC group and non-ABC group. Hence, prospective studies with adequate power are needed to further confirm our findings.

## Conclusions

In a large cohort of Chinese AF patients, ABC integrated management presents a significant association with a reduced risk for all-cause motality and composite outcome of all-cause death, ischemic stroke and intracranial hemorrhage. With higher number of the components of ABC compliance fulfilled, the risk of clinical events was progressively lower.

An increasing number of ABC criteria fulfilled was associated with a progressively lower risk of adverse clinical outcomes.

## Data Availability Statement

The original contributions presented in the study are included in the article, further inquiries can be directed to the corresponding author.

## Ethics Statement

The studies involving human participants were reviewed and approved by the Ethics Committees of Beijing Anzhen Hospital. The patients/participants provided their written informed consent to participate in this study.

## Author Contributions

Y-FW wrote this paper with help of other professors, including making study design, analyzing data, and revising this article. All authors contributed to the article and approved the submitted version.

## Funding

This work was supported by the National Key Research and Development Program of China (Grant Numbers: 2018YFC1312501 and 2017YFC0908803) and the National Natural Science Foundation of China (82103904). The construction of the Chinese Atrial Fibrillation Registry was also supported by grants from Bristol-Myers Squibb, Pfizer, Johnson & Johnson, Boehringer-Ingelheim, and Bayer.

## Conflict of Interest

C-SM has received honoraria from Bristol-Myers Squibb, Pfizer, Johnson & Johnson, Boehringer-Ingelheim, and Bayer for giving lectures. J-ZD has received honoraria from Johnson & Johnson for giving lectures. GL has served as a Consultant and Speaker for BMS/Pfizer, Boehringer Ingelheim, and Daiichi-Sankyo. The remaining authors declare that the research was conducted in the absence of any commercial or financial relationships that could be construed as a potential conflict of interest.

## Publisher's Note

All claims expressed in this article are solely those of the authors and do not necessarily represent those of their affiliated organizations, or those of the publisher, the editors and the reviewers. Any product that may be evaluated in this article, or claim that may be made by its manufacturer, is not guaranteed or endorsed by the publisher.
